# Cerebellar molecular layer interneurons are dispensable for cued and contextual fear conditioning

**DOI:** 10.1038/s41598-020-76729-4

**Published:** 2020-11-17

**Authors:** Katy L. H. Marshall-Phelps, Gernot Riedel, Peer Wulff, Marta Woloszynowska-Fraser

**Affiliations:** 1 Institute of Medical Sciences, University of Aberdeen, Foresterhill, Aberdeen, AB25 2ZD UK; 2 Institute of Physiology, Christian-Albrechts-University Kiel, 24098 Kiel, Germany; 3 Present Address: Centre for Discovery Brain Sciences, Edinburgh, EH16 4SB UK; 4 Present Address: National Institute on Aging, National Institutes of Health, Baltimore, MD 21224 USA

**Keywords:** Cerebellum, Emotion, Neural circuits, Synaptic transmission, Neuroscience, Learning and memory, Consolidation, Fear conditioning

## Abstract

Purkinje cells are the only output cell of the cerebellar cortex. Their spatiotemporal activity is controlled by molecular layer interneurons (MLIs) through GABA_A_ receptor-mediated inhibition. Recently, it has been reported that the cerebellar cortex is required for consolidation of conditioned fear responses during fear memory formation. Although the relevance of MLIs during fear memory formation is currently not known, it has been shown that synapses made between MLIs and Purkinje cells exhibit long term plasticity following fear conditioning. The present study examined the role of cerebellar MLIs in the formation of fear memory using a genetically-altered mouse line (PC-∆γ2) in which GABA_A_ receptor-mediated signaling at MLI to Purkinje cell synapses was functionally removed. We found that neither acquisition nor recall of fear memories to tone and context were altered after removal of MLI-mediated inhibition.

## Introduction

The cerebellum is best known for its role in the coordination and timing of movements. In addition, its function extends beyond that of motor control to several non-motor processes including associative learning^1^ and the regulation of emotional behaviour^2–4^. One example of the latter is the involvement of the cerebellum in the emotion of fear and the generation of fear-related behaviours, which are traditionally attributed to several forebrain structures, and for which the role of the cerebellum and its intrinsic circuitry remains elusive.

Previous studies in humans have confirmed a notable activation of the cerebellum during the mental recall of a fearful episode or following the presentation of sensory stimuli predictive of a painful experience^2,5,6^. In animals, the processes underlying fear memory have been explored using associative fear conditioning, in which a neutral conditioned stimulus (CS), typically a tone, is repeatedly paired with an aversive unconditioned stimulus (US), usually an electric foot shock, until the neutral stimulus comes to elicit a set of defensive conditioned responses (CRs). In the case of rodents, the magnitude of the CR recorded as freezing (absence of movement) is a proxy of acquired fear. While the tone represents a foreground CS, the environmental context, in which the shock was experienced, provides a background CS, and the neuronal pathways underlying these forms of conditioning have been mapped to a large degree^7–10^. A single training session is typically sufficient to elicit robust fear memory, which is retained for long periods of time and can be independently interrogated for either context- or cue-associated fear memories^11^.

**Figure 1:**
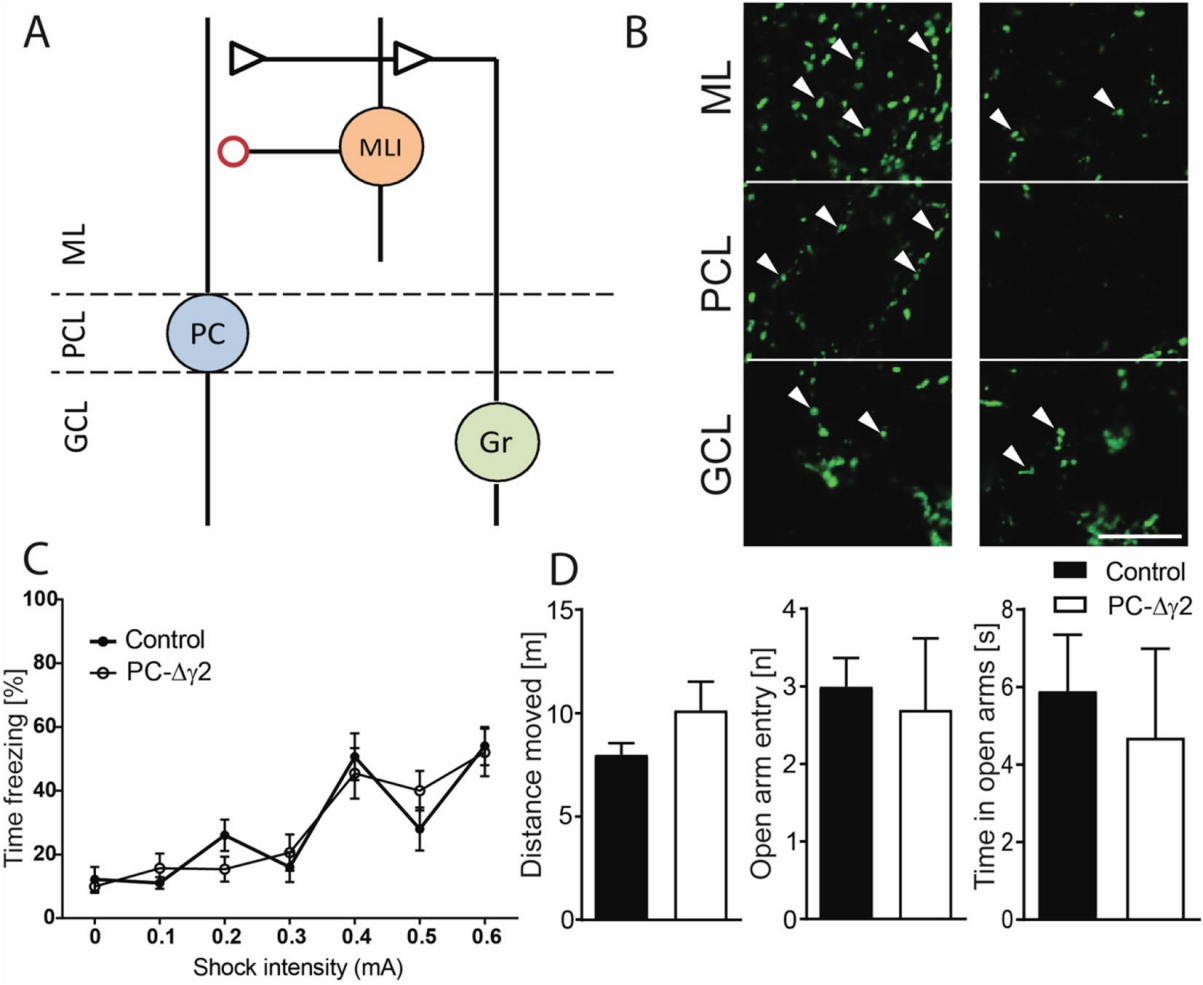
Removal of fast synaptic inhibition from Purkinje cells does not affect pain sensitivity or anxiety. (**A**) Schematic of the cerebellar cortical circuitry studied here. Parallel fibres from granule cells (Gr) provide excitatory input onto Purkinje cells (PCs), but also innervate MLIs, which inhibit PCs. In PC-Δγ2 mice synaptic inhibition between MLIs and PCs (red circle) is disrupted. GCL, granule cell layer; PCL, Purkinje cell layer; ML, molecular layer. (**B**) Immunostaining for the GABA_A_ receptor γ2 sub-unit in sagittal cerebellar sections taken from control (left panel) and PC-∆γ2 (right panel) mice. Arrowheads indicate clusters of γ2 immuno-reactivity. Note the lack of peri-somatic labelling in the PCL and the reduction of labelling in the ML of PC-∆γ2 mice. Residual labelling may derive from e.g. Golgi cells and MLIs. γ2 immuno-reactivity in the GCL is unaltered. Scale bar = 10 μm. (**C**) Pain sensitivity measured as the percentage time spent freezing with increasing shock intensities was unaffected in PC-∆γ2 mice. (**D**) Performance of the animals was tested in the elevated plus maze (EPM). Distance moved, number of open arm entries, and time spent in open arms did not differ between the control and PC-∆γ 2 mice. Data are expressed as mean ± SEM.

Several lines of evidence support a role of the cerebellar cortex in context- and tone-associated fear memories in animals. Reversible inactivation of the vermis following acquisition learning impaired context and cued fear memory up to several days post-training^11^, and intact signalling at parallel fibre to Purkinje cell synapses appears to be critical for recall of cued fear memories^12^. In line with these data, amygdala-dependent post-synaptic plasticity of parallel fibre to Purkinje cell synapses was demonstrated in vermal lobules V and VI following fear conditioning, suggesting that the cerebellar cortex is part of a distributed fear memory network^12–14^. As parallel fibres drive Purkinje cell firing, they also excite molecular layer interneurons (MLIs), comprising stellate and basket cells, and these in turn inhibit Purkinje cell output (Fig. 1A)^15–22^. On the one hand through feed-forward inhibition MLIs sharply curtail EPSPs in Purkinje cells that are excited by the same parallel fibres (on-beam), limiting the time window for summation of excitatory inputs^17^. On the other hand MLIs inhibit Purkinje cells that are located laterally from the active parallel fibres (off-beam, lateral inhibition) and thus modulate the spatial extent of Purkinje cell activity^21^. Accordingly, MLIs control both, the spatial and temporal patterns of Purkinje cell firing. While it is not clear what the functional role of MLI-mediated inhibition is in the context of fear conditioning, MLIs have been implicated in fear memory following the observation of synaptic plasticity at their input from parallel fibers and their outputs onto Purkinje cells after fear conditioning^19,23,24^. This is consistent with the notion that information about the CS and US carried by parallel and climbing fibres reaches MLIs^25^. Accordingly, a functional disconnection between MLIs and Purkinje cells would be expected to compromise the spatial and temporal fidelity of Purkinje cell activity underlying the learning of CS-US associations and their long-term memory. This was investigated here by removal of fast synaptic inhibition onto Purkinje cells in transgenic mice with a Purkinje cell specific deletion of the GABA_A_ receptor γ2 subunit (PC-∆γ2 mice)^20,26^. We found, that neither learning nor short-term or long-term context or cued fear memory were compromised in PC-∆γ2 mice.

## Methods

### Animals

A total of 68 PC-∆γ2 (28 male and 40 female) and 74 control (34 male and 40 female) γ2Lx adult mice (8–14 weeks-old) matched by age were used in this study. PC-∆γ2 and γ2Lx mice were generated as previously described^20,26^. Mice homozygous for γ2Lx were crossed with mice heterozygous for γ2Lx and hemizygous for an L7-Cre transgene^20,27^. γ2Lx mice contain the GABA_A_ receptor γ2 subunit flanked by lox P sites, L7-Cre mice express Cre recombinase under the Purkinje cell specific L7 promoter. γ2Lx/γ2Lx/L7-Cre (PC-∆γ2) and γ2Lx/γ2Lx (control) littermates of the 5th generation were used for all behavioural experiments. Genotypes were confirmed by PCR analysis of DNA from mouse tail/ear biopsies. Animals were group housed (up to 6 per cage) in wire lid cages (Macrolon II) on corn cob bedding and enrichment (paper strips and cardboard tubes) in a controlled facility (temperature 20–22 °C, 60–65% humidity, 17–20 air changes per hour) and ad libitum access to water and food pellets under a 12-h light/dark cycle (lights on at 07:00am). Experiments were carried out in accordance with the European Communities Council Directive (63/2010/EU) and a project licence with ethical approval by the Ethical Review Committee of the University of Aberdeen under the UK Animals (Scientific Procedures) Act (1986). All behavioural testing was performed during the light phase of the cycle.

### Behavioural testing

#### Anxiety tested in the elevated plus maze

Anxiety levels in PC-∆γ2 mice were tested against controls (n = 10 in each group) using the elevated plus maze task. The elevated plus maze was constructed from grey Perspex and consisted of two opposed open arms (35 cm long, 5 cm wide) and two opposed closed arms of identical dimension, but with 17 cm high walls. All arms were perpendicular to one another leaving a central 5 cm square zone. The entire apparatus was raised 40 cm above the bench and placed in a quiet experimental room. Mice were placed into the central zone and allowed to freely explore the maze for 5 min before being returned to their home cage. The X–Y coordinates of each subject (body centroid) were tracked using an overhead camera linked to Ethovision 3.1 Pro software (Noldus IT, Wageningen, Netherlands) at 22 Hz sampling frequency. Arm entries were autoscored by the software if the animal’s body centroid crossed into the respective arm and the time spent in open and closed arms was summated over the total period of exploration. The arena was cleaned with non-perfumed, non-alcoholic wipes in between subjects to remove odour cues.

#### Circadian activity in the home cage—PhenoTyper

To measure circadian activity of the animals, PhenoTyper home-cages (Noldus IT) were utilised in line with the protocol described elsewhere^28,29^. In brief, PhenoTypers of 30 cm × 30 cm floor dimension were equipped with an overhead camera and infra-red lights (for night vision) placed in the lid of the box for long-term and light independent tracking. The front panel also contained both food and water dispensers. Corncob and small amounts of wood shavings were provided as bedding, but no further enrichment was given to aid tracking. Animals (control n = 14, PC-∆γ2 n = 13) were individually placed into a PhenoTyper home cage at ~ 10 a.m. on a Friday, habituated for 3 days and then recorded for 4 consecutive days with free access to food and water. Ambulatory activity was continuously tracked using Ethovision 3.1 Pro (22 Hz sampling rate, body centroid tracking) and path length averaged into hourly bins or a 24 h average. Average activity of the animals during dark (7 p.m.–7 a.m.) and light (7 a.m.–7 p.m.) phases was also contrasted.

#### Motor activity and motor learning—Rotarod

Motor activity of mice was assessed on two automated 4 lane Rotarod’s (UgoBasile, Comerio, Italy). Animals (control n = 13, PC-∆γ2 n = 11) were trained over 3 days, 4 trials per day, in an accelerating rod protocol^30,31^. Trials started with 2 rotations per minute (rpm) and accelerated to the maximum of 45 rpm within a total trial length of 5 min. An inter-trial interval of 2–3 min was applied, and trials were terminated when the subjects fell off or maximum time was achieved. Lane allocation was randomised; the apparatus was cleaned with non-alcoholic wipes between squads/trials. Time spent on the rotating rod during the first 3 trials was recorded as a proxy for motor ability; motor learning was calculated as the progressive improvement in performance and as the difference in performance between trial 12 and trial 1.

#### Fear conditioning experiments

##### Apparatus

All fear conditioning experiments were performed in a TSE fear conditioning system (TSE Systems, Bad Homburg, Germany). The conditioning context consisted of a clear Perspex arena (23 × 23 × 35 cm) with ventilation and lighting provided by an overhead 12 V ceiling light and fan (background noise, 60 dB) placed in a sound attenuating cubicle. Electric shocks of pre-determined duration and frequency were delivered through a grid floor made of stainless-steel rods (diameter = 4 mm, spaced 9 mm apart) connected to a shocker/scrambler unit. A loudspeaker in the distal wall provided the acoustic CS. Movement of the animal in X–Y and Z coordinates was recorded using two rows of infrared beams set at 2 and 6 cm above the grid (beams spaced 14 mm apart). Lack of beam breaks for > 1 s was continuously recorded as freezing and episodes were later summed into respective time bins dependent on the test phase (see below). The chamber was cleaned with non-perfumed, non-alcoholic wipes in between mice and fecal boli were removed from the dust-pan.

##### Pain sensitivity test—shock intensity curve

The same cohort that underwent plus maze exploration was tested for pain sensitivity. Mice (control n = 10, PC-∆γ2 n = 9) were individually placed into the conditioning chamber and left for 120 s before receiving 5 electric foot shocks of increasing intensity (0.1, 0.2, 0.3, 0.4, 0.5 and 0.6 mA) each lasting for 6 s. The shocks were presented with variable inter-stimulus intervals (ranging from 10 to 120 s), during which the levels of freezing were recorded. The mice were then left in the chamber for a further 60 s before being returned to their home cage.

##### Contextual and cued fear conditioning

Three cohorts of PC-∆γ2 (n = 34) and control mice (n = 37) were used to assess fear learning and memory. All cohorts received acquisition training and were then tested for recall at 24hrs (Cohort 1: PC-∆γ2 n = 9; control n = 9), at 10 days (Cohort 2: PC-∆γ2 n = 14; control n = 14), or at 31 days post-training (Cohort 3: PC-∆γ2 n = 11; control n = 14). Prior to fear conditioning, animals were handled for 1–2 min per day for 5 days and habituated to the training chamber for 10 min on the day preceding training. During the habituation session, mice were presented with 12 tones (10 kHz, 70 dB, 6 s) each separated by 30 s intervals. Mice remained in the chamber for a further 60 s before being returned to their home cages. Fear conditioning consisted of a 120 s context exposure (context A) followed by 8 CS tones (3.5 kHz, 70 dB, 6 s) each separated by 30 s and co-terminating with the electric foot shock (US) (0.4 mA, 2 s). Mice were then left undisturbed for a further 60 s in the training context before being returned to their home cages. Freezing (the absence of all movement except respiratory) was continuously monitored during the 120 s context exposure, during the tone/shock pairings including post-shock periods, and for the 60 s following training.

##### Context and cued fear testing

Freezing to context A was measured 24 h, 10 days or 31 days following training without acoustic stimuli or shock presentation. Mice were left for 120 s in the conditioning chamber (context A) before being returned to their home cage. Levels of freezing were measured throughout the 120 s period. We also assessed context discrimination in different contexts (context B) 2 h later. Test chambers were modified, and grid floors replaced by PVC (grey or black) while the transparent walls were covered with black and white inserts. Mice were left for 120 s in contexts B and freezing was recorded. Then, 4 CS tones (3.5 kHz, 70 dB, 6 s) separated by 30 s in the absence of electric foot shocks were administered and freezing was recorded for the tone/interval periods. At the end, a further 60 s elapsed before animals were returned to their home cages.

### Immunohistochemistry

Adult mice were terminated by cervical dislocation and the brains rapidly excised. Brains were immediately frozen on powdered dry ice, wrapped in Parafilm (Pechiney Plastic Packaging) and stored at − 80 °C until sectioning. Brains were sectioned at 14 μm sagittally using a Leica cm1900 cryostat.

Analysis of γ2 sub-unit expression in PC-∆γ2 and control mice was performed as previously described for freshly frozen tissue^32^. Sections were briefly fixed in ice-cold methanol for 30 s before being rinsed and stored in phosphate buffered saline (PBS, pH 7.4). Sections were blocked in PBS containing 10% normal goat serum (NGS) and 0.2% Triton X-100 for 60 min at room temperature and subsequently incubated with the primary antibody overnight at 4 °C diluted 1:1000 in PBS containing 10% normal goat serum (NGS) and 0.2% Triton X-100. The primary antibody was a rabbit polyclonal recognising the GABA_A_R-γ2 sub-unit (Synaptic Systems) and was kindly provided by Dr. Marco Sassoè-Pognetto, University of Turin. Sections were then washed three times in PBS for 10 min at room temperature before being incubated with an Alexa Fluor goat anti rabbit 488 secondary antibody (Invitrogen) at a dilution of 1:1000 in PBS containing 10% NGS and 0.2% Triton X-100 for 60 min at room temperature. Sections were washed three times in PBS for 10 min at room temperature and mounted in Mowiol.

### Statistical analysis

Statistical analysis was performed using Prism 8.0 (GraphPad; US). Upon confirmation of a normal distribution, data are presented as mean ± SEM. Group comparisons were based on one way or two-way Analysis of Variance and the source of significance was explored by planned post-hoc comparisons including Student T-tests with Bonferroni correction. Outliers were researched using the method of Grubbs^33^. All analyses were two-tailed and for the null hypothesis to be accepted, alpha was set to 5%. For simplicity, only significant terms are mentioned in the text.

## Results

### Lack of MLI-mediated inhibition does not affect sensory perception, anxiety or motor performance

Removal of fast synaptic inhibition from Purkinje cells was achieved by crossing mice expressing Cre recombinase under the control of the Purkinje cell specific L7 promoter with mice in which the GABA_A_ receptor γ2 subunit allele was flanked by loxP sites to generate PC-∆γ2 mice. Selective loss of the γ2 subunit from Purkinje cells in PC-∆γ2 mice was confirmed by immunofluorescence. In control mice labelling for the γ2 subunit resulted in strong punctate staining in all layers of the cerebellar cortex, whereas in PC-∆γ2 mice strong staining was found only in the granule cell layer. In the Purkinje cell and molecular layer, which contain Purkinje cell somata and dendrites, respectively, staining was strongly reduced (Fig. 1B). As the γ2 subunit is required for post-synaptic localization and normal conductance of GABA_A_ receptors, its removal in PC-∆γ2 mice causes a loss of synaptic GABA_A_ receptors and MLI-mediated fast synaptic inhibition of Purkinje cells^20,26,34,35^.

**Figure 2:**
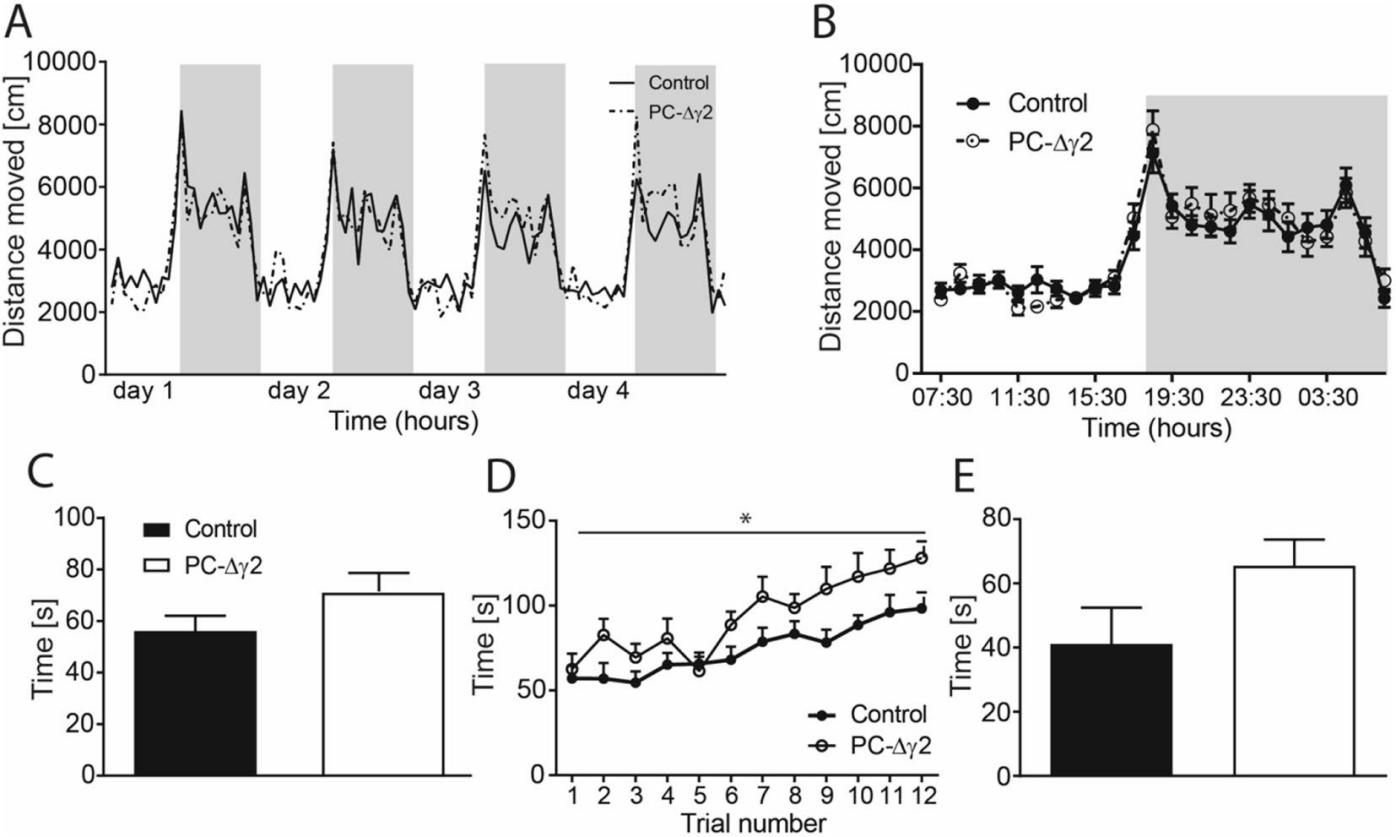
Locomotor activity and motor performance are similar in PC-∆γ2 and control mice. The average distance moved over 4 days (**A**), and the mean distance moved over the 24 h light/dark cycle (**B**) in PhenoTyper boxes did not differ between control and PC-∆γ2 mice. Light background indicates light phase, grey background indicates dark phase. Performance (3 first trials; **C**) on the rotarod was not different between PC-∆γ2 and control mice. However, PC-∆γ2 animals outperformed controls in terms of progressive improvement during rotarod acquisition (**D**). Learning effect (time on the rod on trial 12 minus the time on trial 1) (**E**) on the rotarod was not impaired in PC-∆γ2 mice. Data are expressed as mean ± SEM.

Prior to investigating the role of MLIs in fear learning, we controlled for factors such as pain sensitivity, trait anxiety or locomotor activity, which can affect freezing behaviour during fear conditioning. PC-∆γ2 and littermate control mice showed comparable levels of freezing to unpredictable foot shocks delivered in incrementing intensities (F < 1; Fig. 1C) suggesting that PC-∆γ2 mice present with no sensory deficits related to shock perception (see below). In the elevated plus maze task, both groups of mice showed similar activity, numbers of open arm entries and time spent in the open arms (all t’s < 1.5; p’s > 0.15; Fig. 1D). Similarly, circadian rhythms and overall activity during long term observations in PhenoTyper home cages did not differ between PC-∆γ2 and control animals (main effect of time: *F*(97,2425) = 15.87, *p* < 0.0001; effects with group as factor: F’s < 1; Fig. 2A,B). Finally, motor performance on the rotarod (first 3 trials, Fig. 2C) revealed no differences between the control and PC-∆γ2 mice at the start of testing (t < 1.7), but PC-∆γ2 outperformed controls in terms of progressive improvement of rotarod performance during training (Fig. 2D; main effect of group: *F*(1,242) = 4.38, *p* < 0.05; effect of trial *F*(11,242) = 16.8, *p* < 0.0001; interaction F < 1.4). However, there was no significant difference regarding the overall learning effect (Fig. 2E; P > 0.1).

### Normal context and cued fear conditioning in PC-∆γ2 mice

**Figure 3:**
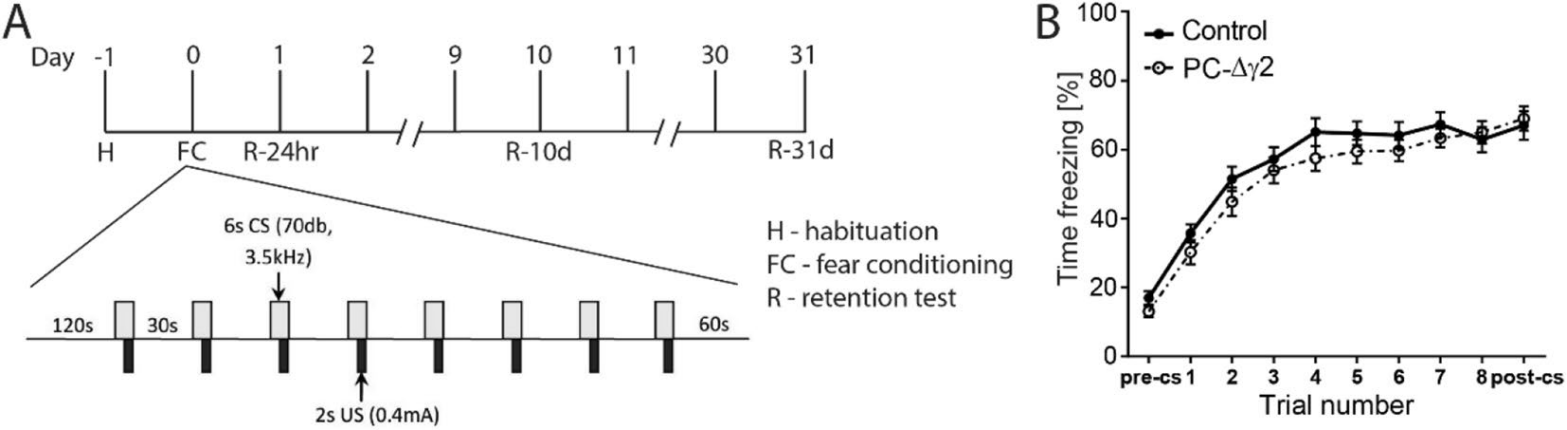
PC-∆γ2 mice show normal fear acquisition. (**A**) Schematic depicting the timeline and protocol used for measuring fear acquisition and retention in control and PC-∆γ2 mice. (**B**) Levels of freezing in control and PC-∆γ2 mice increased (≥ 60% freezing) over the 8 tone-shock pairings. Freezing in PC-∆γ2 animals was not significantly different to controls. Data are expressed as mean ± SEM.

As functional removal of MLI-mediated inhibition did not alter sensory perception to subtle foot shocks, we explored effects on fear learning to a background (context) and foreground (tone) stimulus. Animals were trained in a delayed fear conditioning paradigm adapted from Sacchetti et al.^12^ (Fig. 3A). During fear conditioning the freezing levels for both genotypes increased (≥ 60% of time freezing) with the 8 tone-shock (CS-US) pairings (trial effect: *F*(9.621) = 102.1, *p* < 0.0001; Fig. 3B), but there was no genotype difference (group effects and interaction: F < 1). There was also no difference in freezing during periods prior to onset or after termination of the CS (Fig. 3B).

### Normal background fear recall and context discrimination in PC-∆γ2 mice

Once trained to ceiling performance, we exposed different cohorts of PC-∆γ2 and control mice to contexts without shock at three different time points post acquisition (24 h, 10 days, 31 days) to probe for (i) recall of the conditioning context A (background) and (ii) for another context with explicitly different features to establish context discrimination/stimulus generalisation over time. Theory predicts that if mice are not capable of memorizing context-specific features related to conditioning, stimulus generalisation will take place and mice will also freeze in a different context not previously associated with a foot shock^36^.

**Figure 4:**
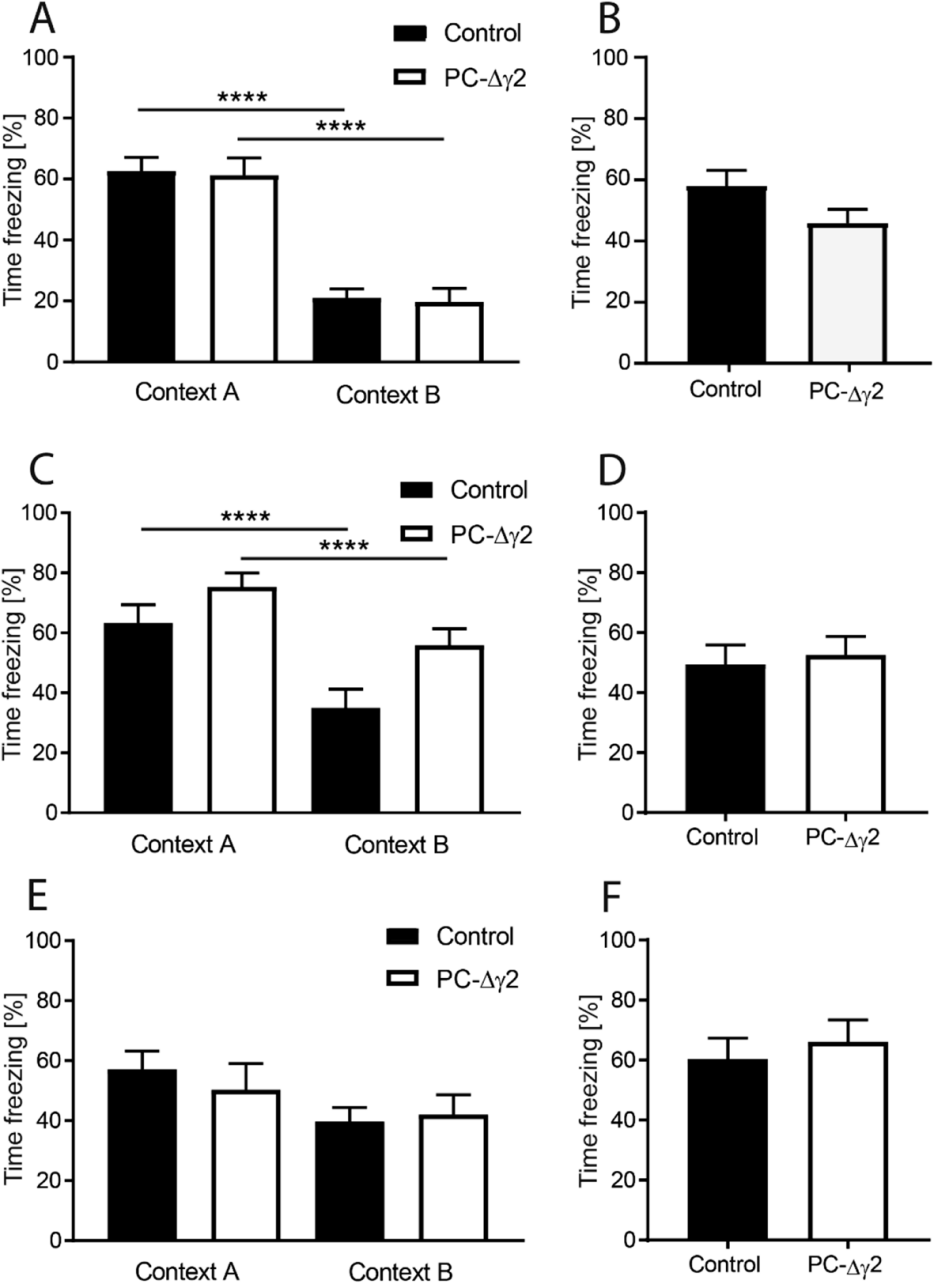
PC-∆γ2 mice show normal fear recall and context discrimination 24 h, 10 and 31 days after fear memory acquisition. Fear memory recall to context and tone was tested at 24 h (**A**,**B**), 10 days (**C**,**D**) and 31 days (**E**,**F**) after training. **(A**,**C**,**E)** Control and PC-∆γ2 mice showed similar levels of freezing in the training context (context A) at 24 h, 10, and 31 days post acquisition indicating normal contextual fear memory recall. Similarly, context discrimination was not affected in PC-∆γ2 mice as both groups were able to distinguish the training context (context A) from the modified context (context B) at all time points measured. Freezing to the cue at 24 h (**B**), 10 days (**D**) and 31 days (**F**) did not differ between control and PC-∆γ2 mice. Data are expressed as mean ± SEM.

Clearly, all cohorts recalled the aversive context and showed about 50–60% freezing time during test at 24 h (Fig. 4A, context A), 10 days (Fig. 4C, context A) and 31 days post-training (Fig. 4E, context A). This occurred similarly in control subjects and PC-∆γ2 mice and there was no difference (t’s < 1.6), indicating normal contextual fear recall over time in PC-∆γ2 mice. Similarly, context discrimination was not different between controls and PC-∆γ2 mice at 24 h post-training (Fig. 4A, contrast of context A versus context B) and both genotypes froze less in the novel context B (no effect of group or interaction: F’s < 1; effect of context *F*(1,16) = 68.98, *p* < 0.0001). Similarly, at 10 days, context discrimination was not significantly different between PC-∆γ2 and control mice (Fig. 4C). This was verified statistically returning main effects of genotype (*F*(1,26) = 5.58, *p* < 0.05) and context *F*(1,26) = 38.23, *p* < 0.0001), but the terms did not interact (F < 1.3). While both groups showed similar freezing to context A, freezing of the PC-∆γ2 animals in context B was significantly higher when compared with control mice (t(26) = 2.62, p < 0.05; Fig. 4C). Pairwise comparison of within-group freezing levels in context A versus B, however, indicated that PC-∆γ2 mice still froze less in context B than in context A (t’s > 5; p’s < 0.01) confirming that PC-∆γ2 animals were able to discriminate the conditioned from the non-conditioned environment.

At 31 days post acquisition both control and PC-∆γ2 animals presented with some discrimination between contexts (Fig. 4E). This was confirmed in a 2-way ANOVA, which returned a main effect of context (F(1,23) = 5; p = 0.03), but no effect of genotype or interaction. These data suggest intact fear memory recall and context discrimination over one month post acquisition.

### Cued fear recall is normal in PC-∆γ2 mice

Tests of cued fear recall were performed at the same time points post acquisition as for background tests (24 h, 10 days, 31 days) in a training-independent context (context B), in which 4 CS tones were replayed and freezing was recorded to these foreground cues. Overall, there was no difference between PC-∆γ2 and control mice at any of the time points post-training (Fig. 4B,D,F; t’s < 1.8; p’s > 0.05). From the data it also appears that forgetting of tone-induced fear over the 31 days is minimal and freezing levels remain high at 50–60% of time.

## Discussion

The present findings demonstrate that cerebellar MLIs are not required for the acquisition or the short- and long-term retrieval of cued or contextual fear memories.

Evidence that the cerebellum is involved in the acquisition and expression of emotional behaviours has been provided by many studies^4,37–42^. Mechanistic insights have come from animal studies investigating the role of the cerebellum in fear conditioning, identifying the cerebellar cortex and the deep cerebellar nuclei as important structures for fear learning and memory^11,43^. During fear learning not only parallel fibre but also MLI synapses onto Purkinje cells undergo plastic changes^12,19^.

To examine the role of MLIs in fear memory, we used a genetic approach to selectively ablate the GABA_A_-γ2 sub-unit from Purkinje cells^20^. While not required for receptor assembly, the γ2 sub-unit is essential for the post-synaptic localisation of GABA_A_ receptors and for normal channel conductance^34,35^. Indeed, we have previously shown that deletion of this sub-unit results in the functional removal of GABA_A_-mediated signalling onto Purkinje cells with no obvious effect on the global and local circuit architecture^20,26^. This approach provides a specific tool for investigating the involvement of MLI signalling in fear learning and memory.

Removal of synaptic inhibition from cerebellar Purkinje cells had no obvious effects on pain sensitivity, anxiety or locomotor activity and did not impair motor performance on a simple rotarod test^20,44^. When tested for cued and contextual fear learning, we found that loss of synaptic inhibition from Purkinje cells did not alter the pace or extent of fear learning. Similarly, we found no deficit in 24 h cued or contextual memory retrieval. These results match the findings reported by Galliano et al.^45^.

To investigate the stability of the acquired fear memory over time, cue- and context associated retention testing was performed in separate cohorts also after 10 days and 31 days following fear acquisition. Each of these retention tests showed similar levels of freezing in both groups indicating intact retention/retrieval of cued and contextual fear memories in PC-∆γ2 mice. To assess the accuracy of the conditioned fear response to the conditioned context, we analysed context discrimination by exposure to a modified environment, which had never been associated with a shock (context B). A generalization of the freezing response over different environments is thought to reflect the forgetting of context-specific features associated with the foot shock and has been shown to increase over time^36,46^. We found that context discrimination was not significantly different between PC-∆γ2 and control animals at any of the three time points tested.

How can we explain the absence of obvious behavioural alterations after disruption of a synapse that has previously been shown to undergo plasticity in the same behavioural paradigm^19^? A simple explanation would be differences in the experimental set up. Whereas Scelfo and colleagues^19^ have detected LTP at MLI to Purkinje cell synapses in young rats (P14 to P16), we have used adult mice (P56-P98) for behavioural evaluation. It is thus possible that interspecies differences or age-related changes in the neuronal circuitry underlying fear learning account for the observed discrepancies (e.g.^47,48^). However, since the genetic approach applied here caused a permanent disruption of the MLI to Purkinje cell synapse, we cannot exclude that over time adaptational processes have compensated for the circuit intervention. One adaptational mechanism that we have previously identified in PC-Δγ2 mice is the reduction in Purkinje cell excitation after parallel fiber stimulation, which helps to maintain E/I balance in Purkinje cells and may be responsible for the unaltered action potential frequencies in vitro and in vivo^20^. In contrast, the temporal fidelity of Purkinje cell responses to parallel fibre stimulation is reduced and their activity patterns are altered due to the loss of feed forward inhibition^20^. This loss of temporal fidelity of Purkinje cell activity appears relevant for behaviours that primarily depend on the cerebellar circuitry and which require precise timing such as adaptation of the vestibulo-ocular reflex or eye blink conditioning^20,49^. However, fear conditioning as assessed in standard protocols may not require this temporal precision in Purkinje cell activity. It thus is conceivable that parallel fibre to Purkinje cell LTP, which is preserved in PC-Δγ2 mice^20^ is sufficient to support a cerebellar role in fear conditioning. Alternatively, as a previous study reported that also chronic disruption of parallel fibre to Purkinje cell LTP does not produce obvious deficits in fear conditioning^45^, it is possible that alterations in other circuits involved in fear conditioning (e.g. amygdala, periaqueductal grey, hypothalamus) compensate for disruptions of the cerebellar circuitry. Thus, although we cannot strictly rule out the involvement of MLIs in fear conditioning we can conclude that MLIs are dispensable for this process.
